# Relationship between Tongue Temperature Estimated by Infrared Thermography, Tongue Color, and Cold-Heat Pathological Patterns: A Retrospective Chart Review Study

**DOI:** 10.1155/2018/6841460

**Published:** 2018-05-27

**Authors:** Seung-Won Baek, Jin-Moo Lee, Young-Bae Park, Young-Jae Park

**Affiliations:** ^1^Department of Biofunctional Medicine and Diagnostics, College of Korean Medicine, Kyung Hee University, Republic of Korea; ^2^Department of Diagnosis and Biofunctional Medicine, Kyung Hee University Hospital at Gangdong, Republic of Korea; ^3^Department of Women's Health Clinic, Kyung Hee University Hospital at Gangdong, Republic of Korea; ^4^Department of Gynecology, College of Korean Medicine, Kyung Hee University, Republic of Korea

## Abstract

Tongue diagnosis is a technique used to determine cold-heat pathologic patterns (CHPPs). Herein, we reviewed electronic medical records of tongue temperature measured using infrared thermography (IRT), tongue color (luminance, green/red, and blue/yellow balance), cold-heat pattern questionnaires (CHPQ), and body temperature for 134 women with gynecological problems (age, 38.97 ± 11.49 years). The temperatures of seven tongue regions (root, center, tip, and both sides of the center and root) were determined, and the effects of age, regional differences, and their correlations with color parameters were examined. Factor analysis was conducted separately with the 10 cold pattern (CP) and 10 heat pattern (HP) items. Tongue temperature showed an age effect (*β*; -0.198 to -0.210) and regional differences (both sides of the root > center and root > tip). Tongue temperature was positively correlated with luminance (r: 0.236-0.246), indicating that a higher temperature was associated with a brighter color. The factor analysis extracted two factors (cold sensitivity-pain and discharge-complexion factors) from the CP items and three factors (heat sensation-pain, discharge-breath, and cold preference-thirst factors) from the HP items. Tongue temperature was negatively correlated with the discharge-complexion factor of CP and the discharge-breath factor of HP (r: -0.171 to -0.203), indicating that a lower tongue temperature may be a consequence of emission of excessive heat in HP and a lower blood perfusion in CP. Body temperature did not correlate with the CHPQ factor scores. In conclusion, tongue temperatures measured using IRT may be a partial indicator of CHPPs.

## 1. Introduction

Different fundamental standards are currently used to identify pathological patterns in East Asian Medicine (EAM). These include the eight-principle identification (EPI) pattern, the viscera and bowels pattern, the six-meridian pattern, the defense (wei), vital energy (qi), nutrient (ying), blood (xue) pattern, and the triple energizer pattern. The EPI pattern is a fundamental classification system consisting of eight components: yin and yang, interior and exterior, cold and heat, and excess and deficiency. Among these components, cold and heat patterns (CP and HP, respectively) are important as they determine an ailment's characteristics and help design a treatment strategy, such as medicine or acupuncture therapy [1, 2].

CP and HP are defined by strict criteria, and various diagnostic techniques have been developed for their assessments. Among these, tongue diagnosis (TD) is considered a key technique because the tongue is connected to the internal organs through meridians, and it is representative of a “cold” or “heat” condition [3]. Typically, TD is performed through visual examination of the color, shape, moisture, movement, and substance of the tongue body, as well as the color, wetness, thickness, and distribution of the tongue coating [3, 4]. However, the results of TD performed with the naked eye are easily affected by environmental factors [3, 4]. Therefore, efforts have been made to design accessible and objective medical devices and algorithms that can reliably quantify TD measurements, such as the ColorChecker [5], digital tongue imaging systems [6], and three-dimensional imaging tongue diagnosis systems [7].

In addition to objective measurements of tongue color, tongue temperature is clinically useful because the tongue has an abundant blood supply, and its surface temperature reflects the internal temperature transferred by blood flow [8–10]. Infrared thermography (IRT) provides an image of the temperature distribution by converting the thermal radiation emitted from the surface of any object or subject to a temperature value [9]. The IRT technique effectively monitors surface temperatures of the human body, which is influenced by factors such as the blood flow near the surface of the body and heat conduction in the deeper blood vessels [11]. Because of its convenience and noninvasiveness, the IRT technique has been used to study some physiological characteristics of the tongue temperature, including age effects and regional differences. Wang et al. [5] reported that the tongue temperature estimated using IRT was associated with age and sex and that there were regional differences in temperature, and Zhu et al. [10, 12] reported that a decrease in tongue temperature might be associated with a decrease in blood perfusion rates in tongues of elderly persons. Jiang et al. [13] reported that the temperature at the root of the tongue was the highest and that it gradually decreased towards the center of the tongue, while the tip of the tongue (TT) had the lowest temperature.

Since tongue temperature is mainly determined by blood flow, CP and HP may be partially related to tongue temperature, as well as tongue color, and the relationship between CP, HP, and tongue temperature may be influenced by the aging effect and regional differences in the tongue. If the tongue temperature simply reflects body temperature, the clinical severity evaluated by CP and HP may be related to both tongue and body temperature. However, few studies have assessed which of the color- or temperature-related parameters of the tongue and body temperature measurements are more indicative of CP and HP and which subscales of the CP and HP may be more strongly associated with tongue temperature. Therefore, in this study, we conducted a retrospective chart review extrapolating data relative to electronic medical recordings of color and temperature parameters of the tongue, body temperature, and quantitative CP and HP scales.

## 2. Methods

### 2.1. Study Design and Participants

**Figure 1:**
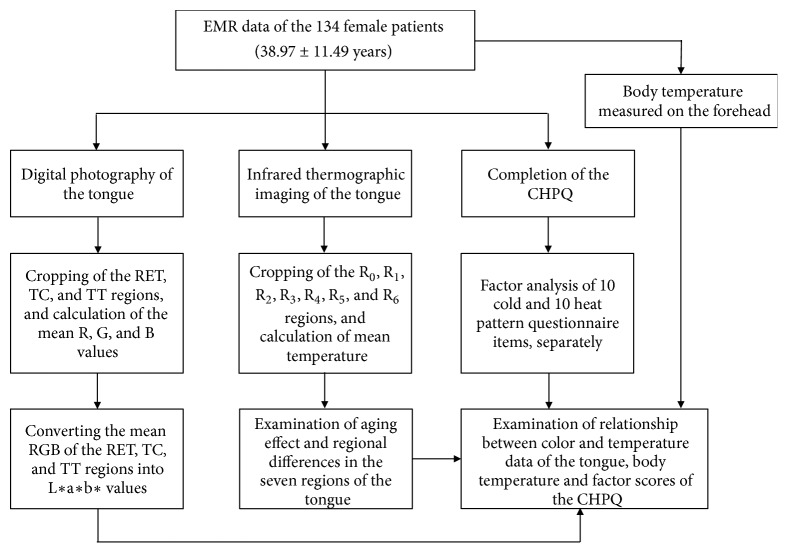
Flow chart of the study. EMR: electronic medical recording; RET: right edge of the tongue; TC: tongue center; TT: tongue tip, R: red; G: green; B: blue; L*∗*: luminance; a*∗*: red/greed balance; b*∗*: yellow/blue balance; CHPQ: cold-heat pattern questionnaire; R_0_: tongue center; R_1_: right side of the tongue center; R_2_: left side of the tongue center; R_3_: tip of the tongue; R_4_: center of the tongue root; R_5_: right side of the tongue root; and R_6_: left side of the tongue root.

The present study was a retrospective chart review. A flowchart summarizing the study design is shown in Figure 1. The electronic medical records (EMRs) of 134 Korean female outpatients that included digital color and IRT images of the patients' tongues were reviewed. Patients had visited the Women's Health Clinic of Kyung Hee University Korean Medical Hospital in Gangdong, Seoul, Korea. The participants were aged between 15 and 71 years (mean: 38.97 ± 11.49 years). In this study, we included outpatients who visited the Women's Health Clinic and underwent four assessments, namely, tongue IRT, tongue photography, cold-heat pattern questionnaire assessment, and body temperature measurement. The study protocol was approved by the Institutional Review Board of the Kyung Hee University Oriental Medical Hospital (KHNMC MD IRB 2011-008).

### 2.2. Cold-Heat Pattern Questionnaire (CHPQ)

The CHPQ was developed by Ryu et al. [14] to estimate the symptoms and signs caused by CP and HP. The questionnaire consists of 20 items (10 cold and 10 heat items) rated on the following 4-point Likert scale: 1 = strongly disagree, 2 = disagree, 3 = agree, and 4 = strongly agree.

### 2.3. Tongue Color Measurement

The subjects' tongues were photographed using a digital camera (D70s; Nikon Co., Tokyo, Japan) and macro-lens (70 mm 1:2.8 dg macro; Sigma Co., Japan) at a resolution of 3000×2000 pixels. Color parameters of the tongue were extracted from EMRs, as described in a previous study [4, 15]. Red (R), green (G), and blue (B) values of the tongue images photographed by a digital camera were calculated using the Picture Color Analyzer software [16] from three cropped regions of interest (ROIs): the right edge of the tongue (RET), the tongue center (TC), and the TT. The average R, G, and B values of the 3 ROIs were converted into the L*∗* (luminance), a*∗* (balance between green [-] and red [+]), and b*∗* (balance between blue [-] and yellow [+]) parameters using Adobe Photoshop software (Adobe Systems Inc., San Jose, CA, USA).

### 2.4. Body and Tongue Temperature Measurement

**Figure 2:**
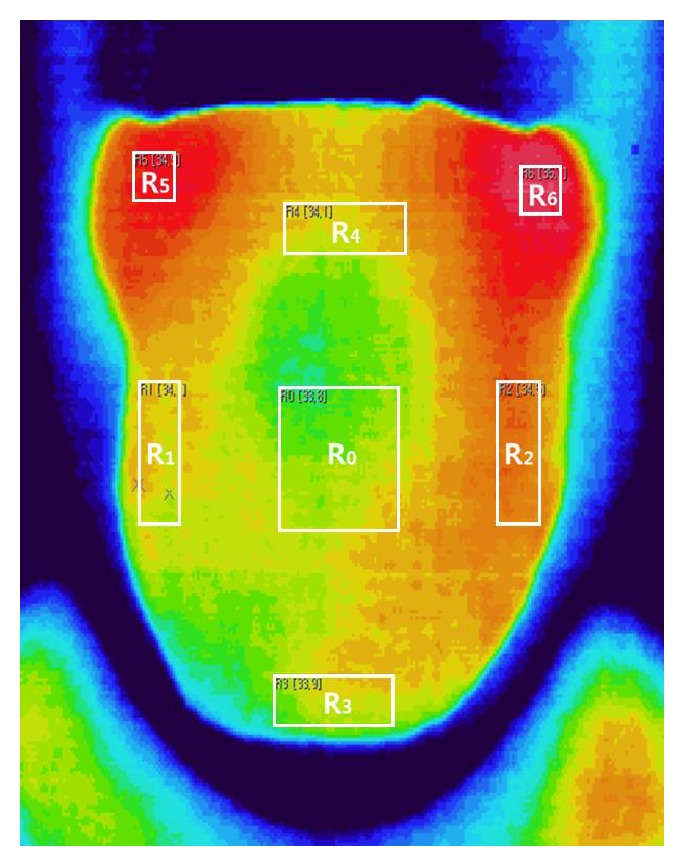
R_0_: tongue center; R_1_: right side of the tongue center; R_2_: left side of the tongue center; R_3_: tip of the tongue; R_4_: center of the tongue root; R_5_: right side of the tongue root; and R_6_: left side of the tongue root.

Body temperatures were measured at the left or right sides of the forehead using an infrared thermometer (Dotory Deluxe Thermoscan FS-100; Hubdic Inc., Anyang, South Korea). Tongue temperature measurement was performed in a room under conditions of controlled temperature (23–24°C) and humidity (40%) according to internationally accepted guidelines [17]. Before the IRT image was captured, outpatients changed into a patient gown and rested for 10 minutes in the testing room to minimize the influence of environment and climate. Subsequently, the patients were asked to undress and wait an additional 15 minutes, without any physical contact, to acclimatize their skin temperature to the room temperature. Patients were asked to expose their tongues entirely in front of the IRT device (digital IRCT-510 camera; Eastwest Co., Seoul, South Korea). The IRCT510 had an accuracy of ±0.08°C, thermal range of −20°C to 250°C, an array size corresponding to a 320 × 240 FPA microbolometer, and a 30° × 40° thermal lens. To avoid congestion of blood at the tip of the tongue, the participants exposed their tongues for less than 5 s. The thermal images were subsequently transferred to a computer, and an examiner processed the images. Typically, five ROIs, that is, the tongue center, right and left sides of the tongue center, tongue tip, and the tongue root, are examined when conducting tongue diagnosis [18]. However, the IRT images of the tongue showed differences in color between the tongue root and both sides of the tongue root during visual inspection. Therefore, seven ROIs, including the right and left sides of the tongue root, were considered in this study to examine whether surface temperature differed between the tongue root and both sides of the tongue root.  Figure 2 shows the seven ROIs as follows: the center (*R*_0_), right side (*R*_1_), and left side (*R*_2_) of the tongue center; the TT (*R*_3_); and the center (*R*_4_), right side (*R*_5_), and left side (*R*_6_) of the tongue root. The mean temperature of each ROI was then analyzed.

### 2.5. Statistical Analysis

A simple linear regression model was used to evaluate the effects of aging on tongue temperature. Among the L*∗*, a*∗*, and b*∗* values, the negative and positive a*∗* and b*∗* values are categorized into green/blue and red/yellow groups. Within each color group, each a*∗* or b*∗* value is considered as an interval value. Therefore, to examine the relationship between tongue color and temperature at R_0_, R_1_, and R_3_, one-way analysis of variance (ANOVA) test or Pearson's correlation coefficient (r) was selected, according to the sample characteristics. ANOVA was also used to evaluate regional differences in tongue temperature. If there were significant differences among the ROIs, Scheffe's post hoc analysis was conducted. On the basis of Scheffe's post hoc analyses, we determined which ROIs of the tongue temperature could be grouped. The 10 items of the CP and the 10 items of the HP were inserted separately in the factor analysis using maximum likelihood extraction. The extracted factors were finally determined by oblique rotation. Factor scores for CP and HP were calculated and saved using the regression method of the factor analysis. Next, Pearson's correlations between the temperature values of the tongue and the factor scores for CP and HP were determined. All statistical analyses were performed using SPSS for Windows Version 21.0 (IBM Corp., Armonk, NY, USA). Values are presented as mean ± standard deviation; a* P *value < 0.05 indicates statistical significance.

## 3. Results

### 3.1. Assessment of the Relationship between Age and Tongue Temperature

Table 1 summarizes the descriptive characteristics of the participants in the study.  Table 2 shows the results of the regression model with age as an independent variable. An age effect was identified for the tongue temperature for all tongue areas (*β* values ranging from -0.198 to -0.215). However, body temperature did not show any age effect. This indicated that the temperature of the tongue surface showed an overall age-related decrease, which was consistent with the results of previous studies [10, 12].

### 3.2. Assessment of the Relationship between Tongue Color Parameters and Tongue Temperature

An assessment of the a*∗* and b*∗* values of all the samples showed that there were no negative values. This indicated that the colors of the three regions (RET, TC, and TT) were yellow and red and that a*∗* and b*∗* values, like the L*∗* value, could be considered as interval values. Therefore, Pearson's correlation values were calculated between temperature and the color parameters of the tongue (Table 3). L*∗* values of the TT were positively correlated with the temperature of the R_0_, R_1_, and R_2_ regions (r: 0.236 to 0.246). This indicated that a higher temperature was associated with a brighter color of the tongue tip.

### 3.3. Assessment of Regional Differences in Tongue Temperature

Table 4 shows the results of the one-way ANOVA comparing regional differences in tongue temperature. Subsequent Scheffe's post hoc analysis revealed that the seven ROIs could be divided into three groups: R_3_ (Group R_A_); R_0_, R_1_, R_2_, and R_4_ (Group R_B_); and R_5_ and R_6_ (Group R_C_).

### 3.4. Assessment of the Relationship between Tongue Temperature and Cold-Heat Pattern Questionnaire Scores

Table 5 summarizes the results of the factor analysis derived from the CHPQ. Two factors were extracted from the CP items and three from the HP items. The total percentage variance of the CP items was 37.0% and that of the HP items was 44.9%. The number for each factor was allocated according to the percentage of variance. Among the CP factors, factor 1 consisted of five cold- and pain-related items (cold sensitivity-pain factor) and factor 2 consisted of five discharge- and complexion-related items (discharge-complexion factor). Among the HP factors, factor 1 consisted of four heat sensation- and pain-related items (heat sensation-pain factor) and factor 2 consisted of four discharge- and respiration-related items (discharge-breath factor). Factor 3 consisted of two cold preference- and thirst-related items (cold preference-thirst factor).

**Figure 3:**
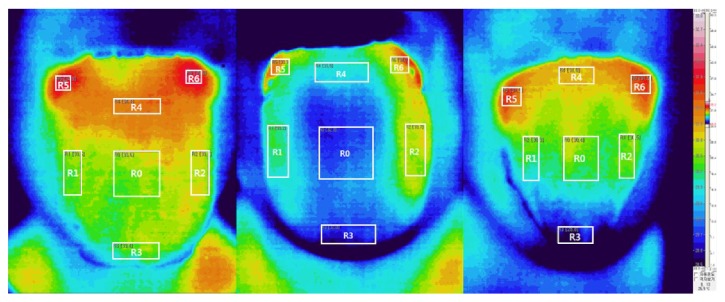
**Left:** This patient showed lower scores for cold factor 2 (8 points) and heat factor 2 (6 points). The temperatures of R_A_, R_B_, and R_C_ (33.4°C, 33.8°C, and 35.0°C, respectively) were higher than average (R_A_: 32.2°C, R_B_: 33.0°C, and R_C_: 34.1°C).** Middle:** This patient recorded higher scores for both cold factor 2 (13 points) and heat factor 2 (8 points). The temperatures at R_A_, R_B_, and R_C_ were lower than average (32.0°C, 33.1°C, and 34.0°C).** Right:** The tongue temperature at R_A_ (28.8°C) was markedly lower than that in the other participants. Moreover, the temperatures at R_B_ and R_C_ were lower than average (30.6°C and 31.5°C, respectively). The patient showed higher scores for cold factor 2 (14 points). R_A_: R_3_; R_B_: R_0_, R_1_, R_2_, and R_4_; R_C_: R_5_ and R_6_. Note that all colors were adjusted using the same thermographic scale, which is shown on the rightmost side of the figure.

The correlations between tongue temperature and cold-heat factor scores are listed in Table 6. Factor 2 (discharge-complexion) scores of the CP were negatively correlated with the R_A_, R_B_, and Rc temperatures (r: -0.195 to -0.197). Similarly, factor 2 (discharge-breath) scores of the HP were negatively correlated with the R_A_, R_B_, and Rc temperatures (r: -0.171 to -0.203). However, body temperature showed no correlation with the CP and HP factor scores. Representative examples of three tongue IRT samples of female patients are shown in Figure 3.

## 4. Discussion

In this study, age was a significant predictor of tongue temperature. This is consistent with the findings of previous studies, which suggest that older individuals have lower tongue temperatures than do younger individuals because their blood perfusion and blood flow rates are lower [10, 12]. Since we did not include a microcirculation test of the TT, it is not clear whether the effect of aging was the result of decreased blood perfusion. However, previous studies have reported that the blood perfusion rate is related to dark tongue colors [19], which is consistent with our results showing that the tongue temperature was negatively related to the brightness of the tongue. Therefore, the effect of aging on tongue temperature in our study may have been partly associated with decreased blood perfusion. In a previous study in which participants were assigned to five groups based on tongue color (red, dark red, purple, pale, or pink) assessed by the naked eye, the tongue temperature was highest in the “red tongue” group [8]. However, our study showed that only the L*∗* parameter on the TT was related to the tongue temperature of all regions. Considering that the TT region is not affected by the tongue coating, our study results suggest that the luminance of the color is more indicative of temperature than tongue redness graded by the naked eye.

An examination of regional differences revealed that the temperature of the tongue tip was lower than that of the root and center regions of the tongue, which is also consistent with previous reports [8, 20]. However, unlike previous studies, we found that temperatures of both sides of the tongue root were higher than those on the other regions. This may be because the vasculature of the lingual artery, which arises from the external carotid artery, runs along both sides of the tongue, but not at the root center [21]. Therefore, we suggest that regional differences in tongue temperature should be considered based on the vasculature of the lingual artery.

The main finding of our study was that two factor scores of the CP and HP were negatively and generally correlated with the temperature of the tongue regions, although the statistical correlations were low (r values ranging from -0.171 to -0.203). Two factors were all discharge-related factors, namely, discharge-complexion factor of the CP and discharge-breath factor of the HP. An increased CP has been reported to decrease the perfusion of blood flow to the lingual artery [10, 12]; thus, it may be natural that an increased CP may have resulted in lower tongue temperatures. However, it seems incongruous that an increased HP was also negatively correlated with lower tongue temperature. A possible explanation for this unexpected result may be that the tongue temperature is a consequence of the emission of excessive heat in HP, as well as a lower blood perfusion in the CP. Irrespective of the CP or HP, discharge-related symptoms related to urine, stool, sputum, and nasal mucus were exclusively correlated with tongue temperature. For this reason, it appears that disturbed discharge conditions may be more representative of cold-heat imbalances than other CP- or HP-related symptoms, and these may be reflected by lower tongue temperatures.

It is also interesting that body temperature measured on the forehead showed no correlation with CP- and HP-related symptoms. A study reported that the normal deep body temperature group (>36.0°C) as well as the low deep body temperature group (<35.5°C) preferred to be warmer, although about half of them said they were warm [22]. Forehead temperature, like body temperature measured under the tongue, is generally accepted to reflect the core temperature, as the superficial temporal artery is located just below the skin and maintains a constant perfusion only under stable conditions [23]. Therefore, this study, consistent with the results of previous studies, suggests that deep temperature may not be associated with one's preference for cold or heat. However, we could not acquire body temperature data measured under the tongue from patient EMRs. Therefore, in future studies, it would be important to examine whether the differences in the relationship between body temperature, tongue temperature, and the CHPQ scores may be simply a result of the measurement site (forehead or tongue) and/or the measuring instrument (thermometer or IRT) used.

In the present study, we observed an age effect on the tongue temperature and regional differences of the tongue, which were consistent with previous findings. Furthermore, we found a significant relationship between the tongue temperature and discharge-related symptoms of tongue CP and HP. Nevertheless, our study had some limitations. First, the data were acquired only from female patients with gynecological conditions and thus have limited generalizability. Second, we could not retrieve body temperature data from the tongue substance. Third, the IRT was blocked by tongue moisture, and the temperature readings may have been affected by the saliva over the tongue surface. Fourth, various disease stages such as acute and chronic stages of infectious or hormonal diseases, which may have affected the relationship between tongue temperature and the clinical severity of CP and HP, were not considered. Fifth, when estimating the tongue temperature, the angle between the tongue and thermal camera was about 70°, not 90°. This may have resulted in a bias in tongue temperature estimation. Sixth, there were differences in the size or shape of the patients' tongues. Moreover, manual cropping of the tongue temperature images may have partially resulted in the bias in the results. Seventh, it is generally accepted that core temperature is modulated according to the menstrual cycle [24]. However, the effect of menstruation on the relationship between tongue temperature and CP and HP could not be examined due to lack of the menstruation data in the EMRs. Further studies are needed to address these limitations.

## 5. Conclusions

In this study, we reviewed the IRT and color data of the tongue, CHPQ scores, and body temperature records of 134 female patients who visited the Women's Health Clinic of the Kyung Hee University Korean Medical Hospital at Gangdong in Seoul, Korea. Our study results showed that the tongue temperature had an age effect (*β*: -0.198 to -0.210), and the temperature differed across different tongue regions (both sides of the tongue root > center and tongue root > TT). Among the five factors evaluated by the CHPQ, two discharge-related factors (discharge-complexion factor of CP and discharge-breath factor of HP) were negatively correlated with the tongue temperature (r: -0.171 to -0.203). In conclusion, our study results suggest that tongue temperature measured using IRT may be a partial indicator of pathological cold and heat patterns, based on discharge-related symptoms.

## Figures and Tables

**Table 1:** Descriptive characteristics of the participants.

Scale	Tongue region	Minimum	Maximum	Mean ± SD
Age (year)		15	71	39.1 ± 11.5
Body temperature (°C)		36.0	36.8	36.3 ± 0.2
Tongue temperature (°C)	R_0_	28.7	35.0	32.7 ± 1.6
	R_1_	29.2	35.4	33.0 ± 1.6
	R_2_	29.2	35.6	33.1 ± 1.6
	R_3_	27.8	34.9	32.2 ± 1.7
	R_4_	28.5	35.4	33.2 ± 1.6
	R_5_	29.3	36.6	34.0 ± 1.5
	R_6_	29.3	36.4	34.1 ± 1.6

Note: R_0_: tongue center; R_1_: right side of the tongue center; R_2_: left side of the tongue center; R_3_: tip of the tongue; R_4_: center of the tongue root; R_5_: right side of the tongue root; and R_6_: left side of the tongue root.

**Table 2:** Simple linear regression model of the effect of aging on the temperatures of the regions of interest.

Scale	B	SE	*β*	t	*P*-value	Adjusted R^2^
Body Temperature (°C)	-0.001	0.001	-0.050	-0.576	0.566	0.050
R_0_ (°C)	-0.029	0.012	-0.210	-2.469	0.015	0.037
R_1_ (°C)	-0.029	0.012	-0.210	-2.473	0.015	0.044
R_2_ (°C)	-0.029	0.012	-0.208	-2.449	0.016	0.036
R_3_ (°C)	-0.027	0.013	-0.178	-2.080	0.039	0.024
R_4_ (°C)	-0.029	0.012	-0.205	-2.411	0.017	0.035
R_5_ (°C)	-0.028	0.011	-0.215	-2.529	0.013	0.046
R_6_ (°C)	-0.027	0.012	-0.198	-2.315	0.022	0.039

Note: B: regression coefficient; SE: standard error; *β*: standardized regression coefficient.

**Table 3:** Pearson's correlation between the tongue temperature and color parameters (L*∗*, a*∗*, and b*∗*).

Scale		RET			TC			TT		
		L*∗*	a*∗*	b*∗*	L*∗*	a*∗*	b*∗*	L*∗*	a*∗*	b*∗*
R_0_	r	-0.017	0.108	-0.026	0.012	0.071	-0.093	0.246^a^	-0.011	-0.134
	*P*-value	0.849	0.216	0.766	0.892	0.418	0.283	0.004	0.897	0.122

R_1_	r	-0.040	0.100	-0.005	0.038	0.004	-0.101	0.243^a^	-0.051	-0.152
	*P*-value	0.650	0.250	0.958	0.659	0.962	0.245	0.005	0.555	0.079

R_3_	r	-0.002	0.086	-0.034	0.009	0.030	-0.079	0.236^a^	-0.024	-0.153
	*P*-value	0.978	0.320	0.700	0.917	0.728	0.364	0.006	0.786	0.077

Note: a*∗*: balance between green (-) and red (+); b*∗*: balance between blue (-) and yellow (+); L*∗*: luminance; RET: the right edge of the tongue; TC: the tongue center; TT: tongue tip; R_0_: tongue center; R_1_: right side of the tongue center; R_3_: tip of the tongue. ^a^*P* < 0.01.

**Table 4:** Temperature differences in each of the regions of interest.

Region (Group)		Mean difference	SE	*P*-value	95% confidence interval	
					Lower bound	Upper bound
R_0_ (R_B_)	R_1_	-0.186	0.196	0.989	-0.881	-0.881
	R_2_	-0.326	0.196	0.839	-1.021	-1.021
	R_3_	0.587	0.196	0.175	-0.109	-0.109
	R_4_	-0.481	0.196	0.419	-1.178	-1.178
	R_5_	-1.251^a^	0.196	< 0.001	-1.947	-1.947
	R_6_	-1.360^a^	0.196	< 0.001	-2.056	-2.056

R_1_ (R_B_)	R_0_	0.185	0.196	0.989	-0.511	-0.511
	R_2_	-0.140	0.196	0.998	-0.836	-0.836
	R_3_	0.772^a^	0.196	0.017	0.076	0.076
	R_4_	-0.296	0.196	0.891	-0.993	-0.993
	R_5_	-1.066^a^	0.196	< 0.001	-1.762	-1.762
	R_6_	-1.175^a^	0.196	< 0.001	-1.871	-1.871

R_2_ (R_B_)	R_0_	0.325	0.196	0.839	-0.372	-0.372
	R_1_	0.140	0.196	0.998	-0.557	-0.557
	R_3_	0.912^a^	0.196	0.001	0.216	0.216
	R_4_	-0.157	0.196	0.996	-0.853	-0.853
	R_5_	-0.926^a^	0.196	0.001	-1.622	-1.622
	R_6_	-1.035^a^	0.196	< 0.001	-1.731	-1.731

R_3_ (R_A_)	R_0_	-0.587	0.196	0.175	-1.284	-1.284
	R_1_	-0.772^b^	0.196	0.017	-1.469	-1.469
	R_2_	-0.912^a^	0.196	0.001	-1.608	-1.608
	R_4_	-1.069^a^	0.196	< 0.001	-1.765	-1.765
	R_5_	-1.838^a^	0.196	< 0.001	-2.534	-2.534
	R_6_	-1.947^a^	0.196	< 0.001	-2.643	-2.643

R_4_ (R_B_)	R_0_	0.481	0.196	0.419	-0.215	1.178
	R_1_	0.296	0.196	0.891	-0.400	0.993
	R_2_	0.157	0.196	0.996	-0.540	0.853
	R_3_	1.069^a^	0.196	< 0.001	0.372	1.765
	R_5_	-0.769^b^	0.196	0.018	-1.466	-0.073
	R_6_	-0.878^a^	0.196	0.003	-1.575	-0.182

R_5_ (R_C_)	R_0_	1.251^a^	0.196	< 0.001	0.555	1.947
	R_1_	1.066^a^	0.196	< 0.001	0.370	1.762
	R_2_	0.926^a^	0.196	0.001	0.230	1.622
	R_3_	1.838^a^	0.196	< 0.001	1.142	2.534
	R_4_	0.769^b^	0.196	0.018	0.073	1.466
	R_6_	-0.109	0.196	0.999	-0.805	0.587

R_6_ (R_C_)	R_0_	1.360^b^	0.196	< 0.001	0.664	2.056
	R_1_	1.175^a^	0.196	< 0.001	0.478	1.871
	R_2_	1.035^a^	0.196	< 0.001	0.339	1.731
	R_3_	1.947^a^	0.196	< 0.001	1.251	2.643
	R_4_	0.878^a^	0.196	0.003	0.182	1.575
	R_5_	0.109	0.15301	0.999	-0.587	0.805

Note: R_0_: tongue center; R_1_: right side of the tongue center; R_2_: left side of the tongue center; R_3_: tip of the tongue; R_4_: center of the tongue root; R_5_: right side of the tongue root; and R_6_: left side of the tongue root; SE: standard error. Groups were categorized according to Scheffe's post hoc analysis: R_A_ = R3; R_B_ = R_0_, R_1_, R_2_, and R_4_; R_C_ = R_5_ and R_6_. ^a^*P* < 0.01; ^b^*P* < 0.05.

**Table 5:** Factor analysis of cold and heat patterns in the cold-heat pattern questionnaire.

Item	Cold Pattern		Item	Heat Pattern		
	Factor 1	Factor 2		Factor 1	Factor 2	Factor 3
Coldness in the limbs	**0.860**	-0.047	Feverish pain	**0.852**	0.053	-0.118
Cold pain	**0.633**	0.339	Body heat	**0.678**	-0.248	0.233
Coldness in the abdomen	**0.606**	0.097	Flushed face and eyes	**0.638**	0.202	-0.077
Aversion to cold	**0.588**	-0.100	Heat in the limbs	**0.584**	0.028	0.221
Preference for heat	**0.538**	-0.024	Thick yellow sputum and nasal mucus	0.025	**0.635**	-0.013
Prolonged voiding of colorless urine	-0.044	**0.574**	Dry stool	0.082	**0.581**	-0.114
Absence of thirst	0.080	**0.514**	Hot breath	0.259	**0.539**	0.124
Loose stools	0.106	**0.497**	Scanty voiding of dark-colored urine	-0.118	**0.468**	0.126
Pale face	0.281	**0.469**	Preference for cold	-0.003	-0.026	**0.789**
Thin clear sputum and nasal mucus	-0.122	**0.328**	Thirst	0.090	0.137	**0.306**
Variance explained (%)	26.67	10.34	Variance explained (%)	27.00	11.02	6.90

Note: bold numbers indicate the greatest factor loading among the three factors and their corresponding items.

**Table 6:** Pearson's correlation between tongue temperature and the scores of the cold-heat pattern questionnaire factors at the R_A_, R_B_, and R_C_ regions.

Group		Cold pattern		Heat pattern		
		Factor 1	Factor 2	Factor 1	Factor 2	Factor 3
Body temperature	r	-0.049	0.037	0.004	-0.081	0.004
	*P-*value	0.575	0.669	0.962	0.354	0.961

R_A_	r	-0.013	**-0.197**	-0.160	**-0.180**	0.009
	*P-*value	0.879	0.023	0.065	0.037	0.916

R_B_	r	-0.071	**-0.195**	-0.161	**-0.203**	0.070
	*P-*value	0.413	0.024	0.062	0.019	0.423

R_C_	r	-0.057	**-0.195**	-0.142	**-0.171**	0.051
	*P-*value	0.515	0.024	0.103	0.048	0.556

Note: R_A_: R_3_; R_B_: R_0_, R_1_, R_2_, and R_4_; R_C_: R_5_ and R_6_; R_0_: tongue center; R_1_: right side of the tongue center; R_2_: left side of the tongue center; R_3_: tip of the tongue; R_4_: center of the tongue root; R_5_: right side of the tongue root; and R_6_: left side of the tongue root. Bold numbers indicate significant correlations.

## Data Availability

The data availability of this study is restricted because sharing the EMR data used in this study may compromise patient privacy.
